# 

**Published:** 1888-09

**Authors:** 


CONTENTS

Vol. 35 No. 9.

| | PAGE. |
|---|---|
| The Summer Vacation | 193 |
| The History of Mind Cure | 194 |
| Washing and Baking Sodas | 200 |
| Adulteration of Food | 204 |
| Resuscitation of those Apparently Drowned | 207 |
| Diphtheria from an Unclean Cellar | 209 |
| Are Corsets a Necessity | 210 |
| Ice Cream | 211 |
| Facts About Honey | 212 |
| Book Notices | 212 |
| Household | 213 |
| Miscellaneous | 215 |

INDEX TO ADVERTISEMENTS OF HALF A PAGE AND OVER.

| | PAGE. |
|---|---|
| Crystal Pepsin Tablets | I |
| Yankee Blade | I |
| Stevensville Mills | II |
| The Tea Cure | II |
| Volapük | III |
| Hollow Suppositories | IV |
| Lactopeptine | V |
| Premiums of Books | VI |
| Premiums of Silver Plate | VII |
| Bovinine | VIII |
| J. Albert Kimball, D. D. S. | IX |
| Rio Chemical Co. | X |
| Page & Co. | XI |

